# Supplementary figures and images for: Persistent homology analysis of type 2 diabetes genome-wide association studies in protein–protein interaction networks
Source: Front Genet. 2023 Sep 26;14:1270185. doi: 10.3389/fgene.2023.1270185 (PMC10562725; doi:10.3389/fgene.2023.1270185)

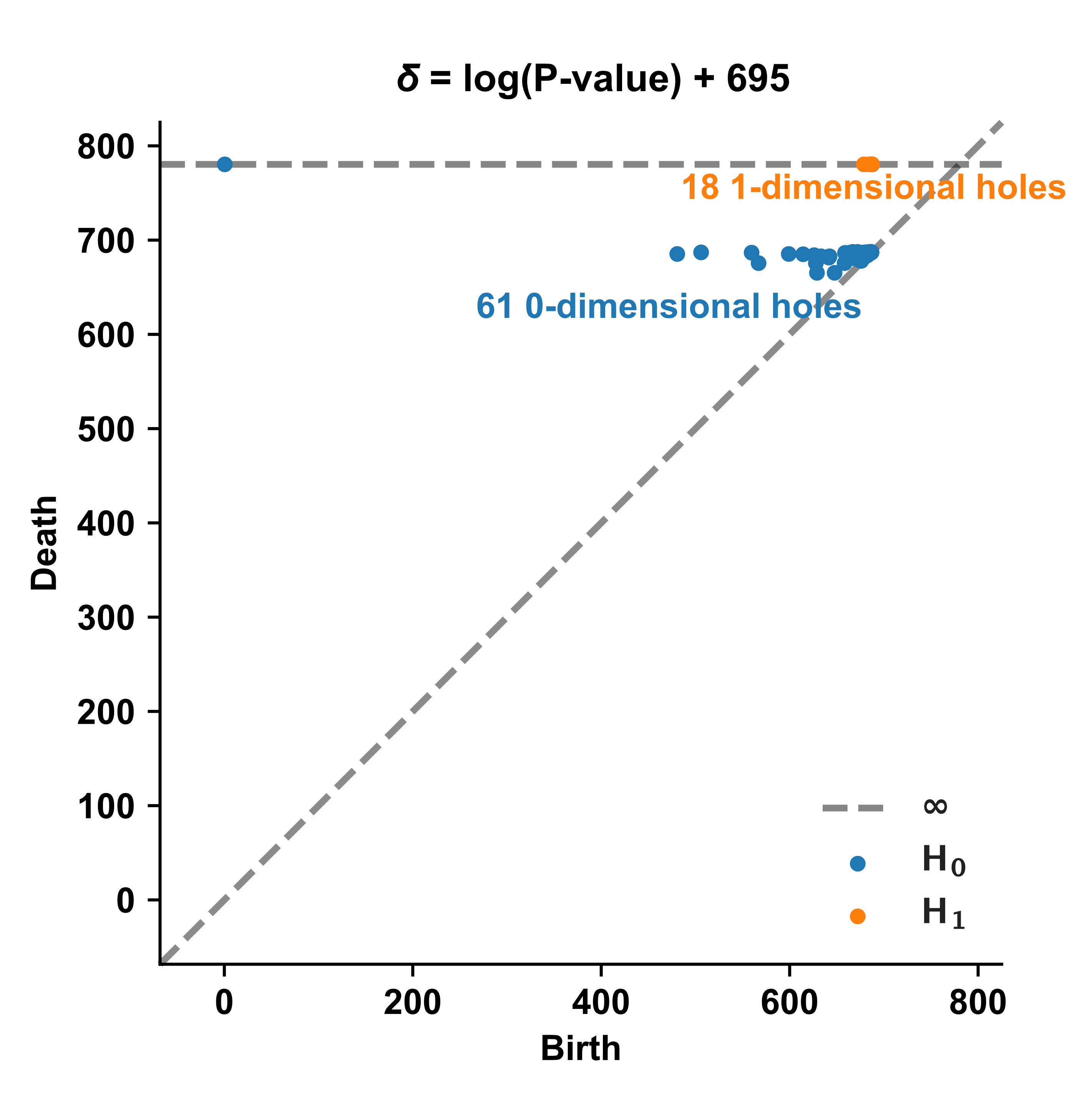

Supplement: Supplementary file 1 [file Image1.PNG]
